# Supplementary material for: Group B Streptococcus adaptation promotes survival in a hyperinflammatory diabetic wound environment
Source: Sci Adv. 2022 Nov 11;8(45):eadd3221. doi: 10.1126/sciadv.add3221 (PMC9651866; doi:10.1126/sciadv.add3221)
Supplement: Supplementary file 1 — Figs. S1 to S4 Tables S1 to S7 References [file sciadv.add3221_sm.pdf]

Science Advances
AAAS

Supplementary Materials for

Group B *Streptococcus* adaptation promotes survival in a hyperinflammatory diabetic wound environment

Rebecca A. Keogh *et al.*

Corresponding author: Kelly S. Doran, kelly.doran@cuanschutz.edu;
Alexander R. Horswill, alexander.horswill@cuanschutz.edu

Sci. Adv. **8**, eadd3221 (2022)
DOI: 10.1126/sciadv.add3221

This PDF file includes:

Figs. S1 to S4
Tables S1 to S7
References

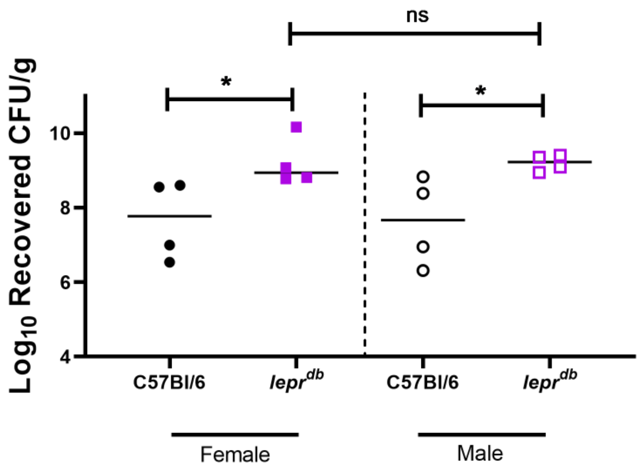

Fig. S1. Murine Model of GBS Diabetic Wound Infection in Female and Male Mice. (A) CFU recovered from wounds of non-diabetic and diabetic mice after GBS infection. All animal infections proceeded for four days with three days under adhesive and sacrifice 24 h after adhesive removal. Significance determined by Mann–Whitney U test; *$p<.05$.

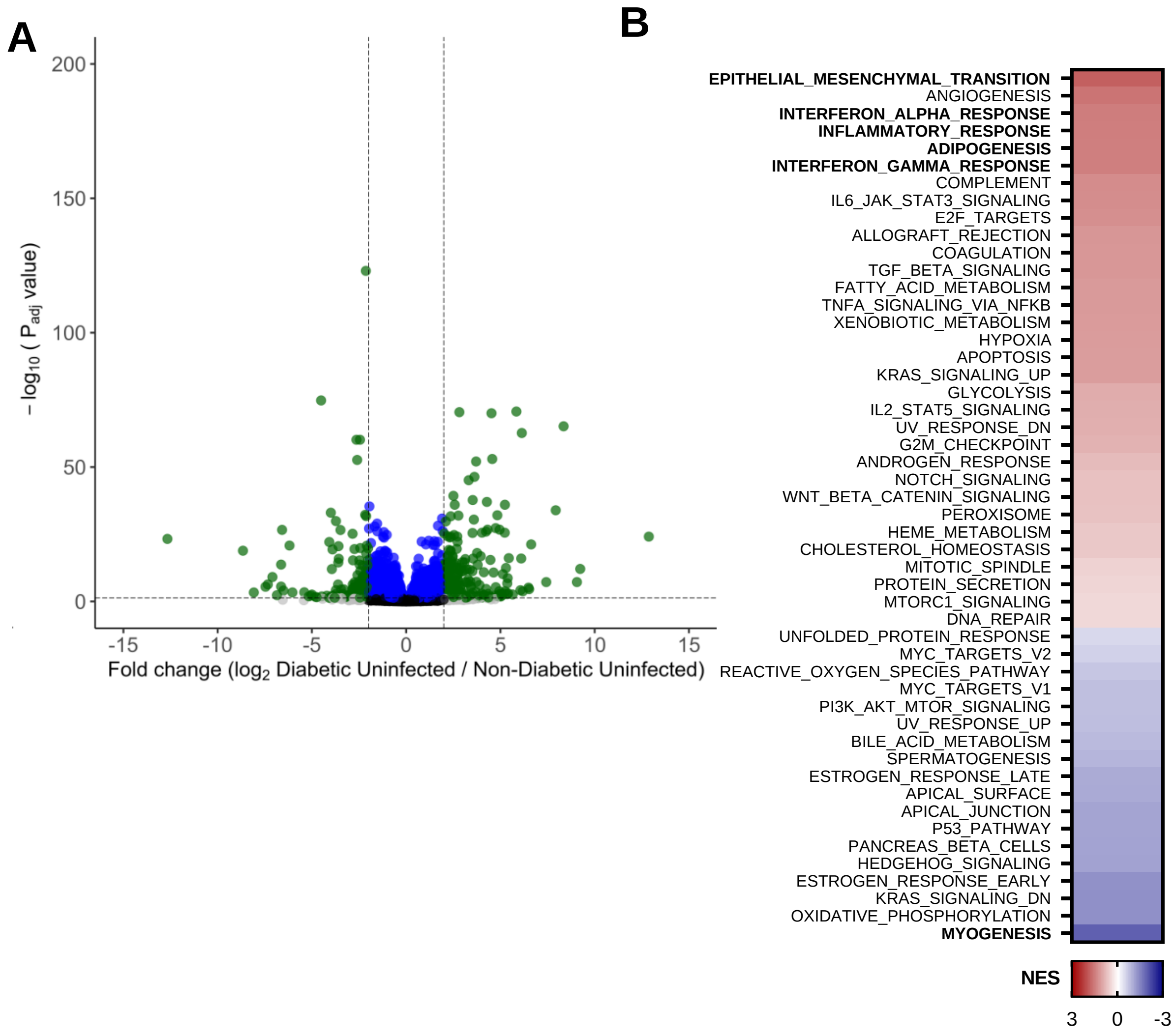

Fig. S2. Murine transcriptome in non-diabetic uninfected vs. diabetic uninfected comparison. (A) Volcano plot of differentially expressed genes. (B) GSEA of pathways enriched in diabetic wounds. Significant pathways are in bold. NES presented as a heat map with highly enriched pathways in red.

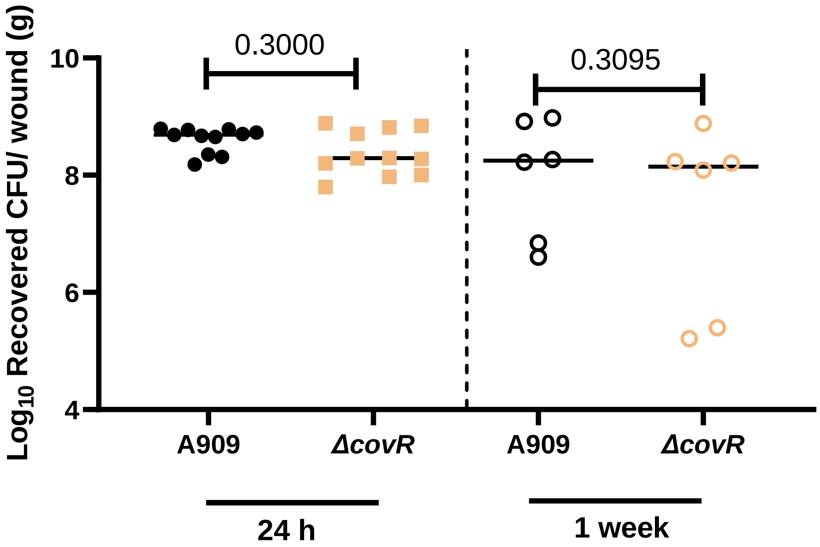

Fig. S3. Analysis of a Δ*covR* mutant in diabetic wound infection. CFU recovered from wounds of diabetic mice after GBS infection. Animal infections proceeded for four or 11 days with three days under adhesive and sacrifice 24 h or 1 week after adhesive removal. Significance determined by Mann–Whitney U test.

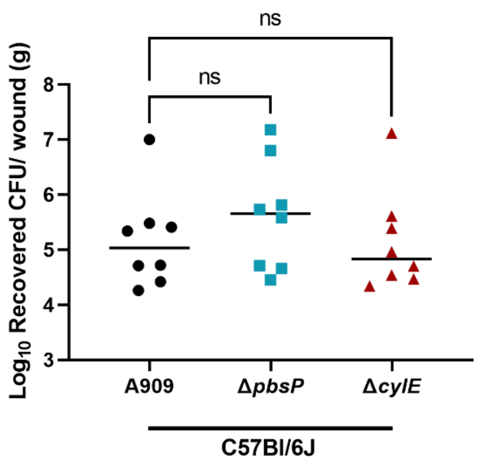

Fig. S4. Analysis of a Δ*pbsP and* Δ*cylE* mutant in non-diabetic wound infection. CFU recovered from wounds of diabetic mice after GBS infection. Animal infections proceeded for four days with three days under adhesive and sacrifice 24 h after adhesive removal. Significance determined by One-way ANOVA with comparison to A909.

Serotype	Ia	Ib	II	III	V	Undetermined
Distribution	**9/27 (33.33%)**	**2/27 (7.41%)**	**6/27 (22.22%)**	**3/27 (11.11%)**	**6/27 (22.22%)**	**1/27 (3.7%)**
**Strain numbers**	112[C]	503[I]	69[C]	64[C]	98[C]	734[I]
	836[I]	1418[I]	85[C]	113[C]	103[C]	
	884[I]		157[C]	361[I]	130[C]	
	982[I]		330[I]		809[I]	
	1052[I]		607[I]		914[I]	
	1394[I]		1962[I]		1101[I]	
	1463[I]					
	1852[I]					
	2000[I]					

Isolates obtained from Colorado are designated with a [C] and isolates from Iowa an [I].

Table S1. Clinical isolate serotypes.

Gene ID	DAVID Description	Diabetic/ non-diabetic log2 FC	padj
**Neutrophil degranulation**			
Ear1	eosinophil-associated, ribonuclease A family, member 1(Ear1)	2.33	1.24E-04
Olfm4	olfactomedin 4(Olfm4)	3.87	6.29E-12
Acaa1b	acetyl-Coenzyme A acyltransferase 1B(Acaa1b)	3.88	6.99E-37
Gpr84	G protein-coupled receptor 84(Gpr84)	2.32	7.57E-06
Hp	haptoglobin(Hp)	2.71	3.74E-23
Slco4c1	solute carrier organic anion transporter family, member 4C1(Slco4c1)	2.12	6.95E-07
Prg2	proteoglycan 2, bone marrow(Prg2)	4.27	1.11E-07
Lrg1	leucine-rich alpha-2-glycoprotein 1(Lrg1)	2.10	4.08E-19
Rab9b	RAB9B, member RAS oncogene family(Rab9b)	2.23	1.85E-02
Mmp8	matrix metallopeptidase 8(Mmp8)	3.82	3.85E-12
Pglyrp1	peptidoglycan recognition protein 1(Pglyrp1)	3.12	2.05E-11
Elane	elastase, neutrophil expressed(Elane)	5.16	1.51E-06
Krt8	keratin 8(Krt8)	3.49	1.23E-04
Prtn3	proteinase 3(Prtn3)	2.93	1.41E-07
Camp	cathelicidin antimicrobial peptide(Camp)	4.49	1.52E-16
Mmp25	matrix metallopeptidase 25(Mmp25)	2.72	2.16E-09
Itgam	integrin alpha M(Itgam)	4.26	1.06E-02
Abca13	ATP-binding cassette, sub-family A (ABC1), member 13(Abca13)	3.64	5.46E-15
Lcn2	lipocalin 2(Lcn2)	3.25	2.54E-30
Cd36	CD36 molecule(Cd36)	3.38	5.74E-49
Itgad	integrin, alpha D(Itgad)	4.75	5.34E-45
Ctsg	cathepsin G(Ctsg)	8.44	3.39E-08
Ms4a3	membrane-spanning 4-domains, subfamily A, member 3(Ms4a3)	5.30	1.12E-03
Mgam	maltase-glucoamylase(Mgam)	2.63	1.45E-07
Epx	eosinophil peroxidase(Epx)	3.31	1.40E-03
Mpo	myeloperoxidase(Mpo)	4.41	4.40E-05
Cd177	CD177 antigen(Cd177)	5.14	3.46E-20
**Activation of Matrix Metalloproteases**			
Mmp25	matrix metallopeptidase 25(Mmp25)	2.72	2.16E-09
Ctsg	cathepsin G(Ctsg)	8.44	3.39E-08
Mmp8	matrix metallopeptidase 8(Mmp8)	3.82	3.85E-12
Tpsab1	tryptase alpha/beta 1(Tpsab1)	3.69	1.60E-14
Elane	elastase, neutrophil expressed(Elane)	5.16	1.51E-06
**Antimicrobial peptides**			
Ear1	eosinophil-associated, ribonuclease A family, member 1(Ear1)	2.33	1.24E-04
Prtn3	proteinase 3(Prtn3)	2.93	1.41E-07
Ctsg	cathepsin G(Ctsg)	8.44	3.39E-08
Clec10a	C-type lectin domain family 10, member A(Clec10a)	2.02	3.03E-17
Camp	cathelicidin antimicrobial peptide(Camp)	4.49	1.52E-16
Pglyrp1	peptidoglycan recognition protein 1(Pglyrp1)	3.12	2.05E-11
Elane	elastase, neutrophil expressed(Elane)	5.16	1.51E-06
Lcn2	lipocalin 2(Lcn2)	3.25	2.54E-30
**Formation of the cornified envelpoe/ keratinization**			
Krt35	keratin 35(Krt35)	-5.82	2.10E-03
Klk13	kallikrein related-peptidase 13(Klk13)	-2.17	1.97E-20
Krt79	keratin 79(Krt79)	-2.14	5.76E-06
Lelp1	late cornified envelope-like proline-rich 1(Lelp1)	-2.25	2.89E-02
Krt71	keratin 71(Krt71)	-7.27	8.43E-06
Sprr3	small proline-rich protein 3(Sprr3)	-2.46	1.29E-02
Lce1b	late cornified envelope 1B(Lce1b)	-2.18	4.53E-05
Krt75	keratin 75(Krt75)	-3.10	7.08E-63
Casp14	caspase 14(Casp14)	-2.53	5.33E-06
Pcsk6	proprotein convertase subtilisin/kexin type 6(Pcsk6)	-2.31	1.07E-21
Lce1f	late cornified envelope 1F(Lce1f)	-2.02	7.08E-05
Krt73	keratin 73(Krt73)	-6.34	2.07E-03
Klk12	kallikrein related-peptidase 12(Klk12)	-2.09	8.60E-13
Krt28	keratin 28(Krt28)	-4.51	1.26E-04
Krt27	keratin 27(Krt27)	-7.76	8.43E-06
Krt25	keratin 25(Krt25)	-8.34	1.47E-04
Lce6a	late cornified envelope 6A(Lce6a)	-2.09	1.60E-03
Krt82	keratin 82(Krt82)	-4.69	9.87E-03
Flg	filaggrin(Flg)	-3.73	1.93E-06
Cdsn	corneodesmosin(Cdsn)	-2.13	2.08E-11
Ivl	involucrin(Ivl)	-3.08	2.16E-11
Rptn	repetin(Rptn)	-3.27	3.10E-14
Klk8	kallikrein related-peptidase 8(Klk8)	-2.12	7.20E-13
Spink5	serine peptidase inhibitor, Kazal type 5(Spink5)	-2.05	3.10E-22
Klk5	kallikrein related-peptidase 5(Klk5)	-2.05	1.41E-07
**FGFR1 ligand binding and activation**			
Flg	filaggrin(Flg)	-3.73	1.93E-06
Fgf4	fibroblast growth factor 4(Fgf4)	-2.49	3.77E-02

Table S2. Murine transcriptome in diabetic infected vs. non-diabetic infected comparison. Select genes and pathways.

Gene ID	DAVID Description	Diabetic infected/ uninfected log2 FC	padj
**Neutrophil Degranulation**			
Ear1	eosinophil-associated, ribonuclease A family, member 1(Ear1)	2.17	8.85E-04
Tarm1	T cell-interacting, activating receptor on myeloid cells 1(Tarm1)	2.29	6.11E-05
Olfm4	olfactomedin 4(Olfm4)	3.04	4.43E-07
Gpr84	G protein-coupled receptor 84(Gpr84)	2.70	7.45E-07
S100a8	S100 calcium binding protein A8 (calgranulin A)(S100a8)	2.73	1.61E-09
Slco4c1	solute carrier organic anion transporter family, member 4C1(Slco4c1)	2.05	6.44E-06
S100a9	S100 calcium binding protein A9 (calgranulin B)(S100a9)	2.55	2.91E-10
Prg2	proteoglycan 2, bone marrow(Prg2)	4.50	2.89E-07
Mmp8	matrix metallopeptidase 8(Mmp8)	4.73	3.51E-17
Pglyrp1	peptidoglycan recognition protein 1(Pglyrp1)	3.98	8.18E-17
Elane	elastase, neutrophil expressed(Elane)	5.93	1.45E-06
Prtn3	proteinase 3(Prtn3)	2.77	2.91E-06
Camp	cathelicidin antimicrobial peptide(Camp)	5.72	3.39E-20
Mmp25	matrix metallopeptidase 25(Mmp25)	3.15	1.74E-11
Abca13	ATP-binding cassette, sub-family A (ABC1), member 13(Abca13)	4.35	1.15E-19
Lcn2	lipocalin 2(Lcn2)	4.41	1.71E-54
Gzmb	granzyme B(Gzmb)	2.33	1.59E-07
Cxcl1	chemokine (C-X-C motif) ligand 1(Cxcl1)	2.20	1.79E-06
Fpr1	formyl peptide receptor 1(Fpr1)	2.79	5.57E-07
Cxcr1	chemokine (C-X-C motif) receptor 1(Cxcr1)	2.42	1.66E-05
Ctsg	cathepsin G(Ctsg)	3.08	1.88E-03
Ms4a3	membrane-spanning 4-domains, subfamily A, member 3(Ms4a3)	7.25	5.06E-04
Mgam	maltase-glucoamylase(Mgam)	3.26	3.34E-10
Epx	eosinophil peroxidase(Epx)	4.37	1.09E-03
Mpo	myeloperoxidase(Mpo)	6.35	3.09E-08
Cd177	CD177 antigen(Cd177)	3.91	2.95E-11
**Signaling by Interleukins**			
Nos2	nitric oxide synthase 2, inducible(Nos2)	4.69	2.21E-10
Il12b	interleukin 12b(Il12b)	2.50	1.12E-02
Ccl5	chemokine (C-C motif) ligand 5(Ccl5)	3.29	2.22E-10
Il12a	interleukin 12a(Il12a)	2.68	3.60E-04
Il27	interleukin 27(Il27)	2.77	1.32E-06
Saa1	serum amyloid A 1(Saa1)	3.61	2.01E-21
Prtn3	proteinase 3(Prtn3)	2.77	2.91E-06
Csf2	colony stimulating factor 2 (granulocyte-macrophage)(Csf2)	3.04	2.99E-02
Lcn2	lipocalin 2(Lcn2)	4.41	1.71E-54
Ebi3	Epstein-Barr virus induced gene 3(Ebi3)	2.58	8.05E-09
Mpl	myeloproliferative leukemia virus oncogene(Mpl)	2.06	2.97E-02
Gzmb	granzyme B(Gzmb)	2.33	1.59E-07
Cxcl1	chemokine (C-X-C motif) ligand 1(Cxcl1)	2.20	1.79E-06
Fpr1	formyl peptide receptor 1(Fpr1)	2.79	5.57E-07
Ctsg	cathepsin G(Ctsg)	3.08	1.88E-03
**Formation of a fibrin clot**			
Gp9	glycoprotein 9 (platelet)(Gp9)	2.12	2.36E-04
Fga	fibrinogen alpha chain(Fga)	6.12	1.58E-03
Prtn3	proteinase 3(Prtn3)	2.77	2.91E-06
Cd177	CD177 antigen(Cd177)	3.91	2.95E-11
**Striated muscle contraction**			
Myh8	myosin, heavy polypeptide 3, skeletal muscle, embryonic(Myh3)	-2.79	4.54E-07
Myh3	myosin, heavy polypeptide 8, skeletal muscle, perinatal(Myh8)	-2.65	3.00E-06
Actc1	actin, alpha, cardiac muscle 1(Actc1)	-2.86	3.17E-04
**Myogenesis**			
Ctnna2	catenin (cadherin associated protein), alpha 2(Ctnna2)	-2.24	3.59E-06
Myf5	myogenic factor 5(Myf5)	-2.41	5.88E-03

Table S3. Murine transcriptome in diabetic infected vs. diabetic uninfected comparison. Select genes and pathways.

Locus Tag (CJB111)	Gene name	Description	Diabetic/ Non-diabetic	padj
ID870_08840	*hrtB*	FtsX-like permease family protein	254.88	2.45E-08
ID870_01095		universal stress protein	47.59	2.19E-30
ID870_08835	*hrtA*	ABC transporter ATP-binding protein	37.58	2.69E-04
ID870_05815	*fetB*	iron export ABC transporter permease subunit FetB	14.81	4.20E-06
ID870_02600	*pilB*	PI-2a pilus major subunit PilB	12.90	3.04E-03
ID870_03655		LrgB family protein	10.85	7.81E-03
ID870_08490	*lrgA*	CidA/LrgA family protein	10.12	4.31E-10
ID870_08485	*lrgB*	antiholin-like protein LrgB	8.67	1.03E-07
ID870_03740	*tpx*	thiol peroxidase	8.55	3.59E-25
ID870_08825	*hssS*	HAMP domain-containing histidine kinase	6.67	1.68E-12
ID870_05980		serine hydrolase	6.48	2.03E-02
ID870_08830	*hssR*	response regulator transcription factor	6.40	2.29E-04
ID870_02595	*pilA*	PI-2a pilus adhesin PilA	5.77	8.47E-03
ID870_06030		SpaH/EbpB family LPXTG-anchored major pilin	5.53	1.61E-02
ID870_03660		CidA/LrgA family protein	4.71	2.12E-02
ID870_00130	*phoP*	response regulator transcription factor	-3.16	2.26E-03
ID870_02090	*nikE*	ABC transporter ATP-binding protein	-3.17	4.95E-04
ID870_00205		sensor histidine kinase	-3.18	2.51E-08
ID870_08645	*adcC*	metal ABC transporter ATP-binding protein	-3.41	2.16E-08
ID870_05910	*cylJ*	cylJ protein	-3.64	2.34E-08
ID870_00210		response regulator	-3.67	6.26E-05
ID870_04175		WXG100 family type VII secretion target	-3.72	4.44E-07
ID870_02080	*nikC*	ABC transporter permease	-3.76	1.52E-06
ID870_02660		iron ABC transporter permease	-3.79	6.61E-03
ID870_04785		pneumococcal-type histidine triad protein	-4.00	6.37E-07
ID870_07205		S8 family serine peptidase	-4.09	4.67E-09
ID870_02700		ferredoxin	-4.13	3.45E-12
ID870_02075	*nikB*	ABC transporter permease	-4.39	1.18E-06
ID870_03375	*hylB*	hyaluronate lyase	-4.42	5.51E-12
ID870_02070	*nikA*	nickel ABC transporter, nickel/metallophore periplasmic binding protein	-4.67	7.88E-07
ID870_05425		LPXTG cell wall anchor domain-containing protein	-5.04	2.40E-07
ID870_10190		bacteriocin immunity protein	-5.22	3.65E-08
ID870_00255	*shtII*	pneumococcal-type histidine triad protein	-5.56	4.44E-07
ID870_09365		sugar ABC transporter permease	-5.60	5.04E-06
ID870_03205	*lmb*	metal ABC transporter substrate-binding lipoprotein/laminin-binding adhesin Lmb	-5.79	1.43E-10
ID870_00250	*adcAII*	zinc ABC transporter substrate-binding protein	-5.96	1.54E-05
ID870_03210	*sht*	pneumococcal-type histidine triad protein	-6.47	1.26E-18
ID870_07705		PTS sugar transporter subunit IIB	-11.84	2.62E-09
ID870_07700		PTS sugar transporter subunit IIC	-14.29	8.15E-40
ID870_00580		PTS sugar transporter subunit IIC	-19.83	2.09E-44

Table S4. Select GBS transcripts with altered regulation in diabetic wounds vs. non-diabetic.

Category	AA Position	Nucleotide position	Substitution
**AA Substitution**	G38		Gly-to-Glu
	A96		Ala-to-Val
	R66		Arg-to-Cys
	G61		Gly-to-Asp
	D53		Asp-to-His
	R67		Arg-to-Pro
	R67		Arg-to-Cys
	A96		Ala-to-Val
**Nucleotide deletion**		387	Deletion of A
		169	Deletion of T
	V31		Deletion of Val
**Insertion/deletion**		123	Multiple nucleotides deleted
		35	Multiple nucleotides inserted

Table S5. Select GBS transcripts with altered regulation in diabetic wounds vs. non-diabetic.

Strain	strain type	Antibiotic resistance	Reference
CJB111	clinical isolate		81
A909	clinical isolate		82
COH1	clinical isolate		83
A909 ΔpbsP	mutant	Cm 3	65
A909 ΔpbsP::pbsP	complement	Cm 3, Spec 100	65
A909 ΔcylE	mutant	Cm 2	51
A909 ΔcovR	mutant	Cm 3	36

Table S6. Strains used in this study.

**Primer Name**	**Sequence 5’ → 3’**
Serotype Ia Forward	GGTCAGACTGGATTAATGGTATGC
Serotype Ia Reverse	GTAGAAATAGCCTATATACGTTGAATGC
Serotype Ib Forward	TAAACGAGAATGGAATATCACAAACC
Serotype Ib Reverse	GAATTAACTTCAATCCCTAAACAATATCG
Serotype II Forward	GCTTCAGTAAGTATTGTAAGACGATAG
Serotype II Reverse	TTCTCTAGGAAATCAAATAATTCTATAGGG
Serotype III Forward	TCCGTACTACAACAGACTCATCC
Serotype III Reverse	AGTAACCGTCCATACATTCTATAAGC
Serotype IV Forward	GGTGGTAATCCTAAGAGTGAACTGT
Serotype IV Reverse	CCTCCCCAATTTCGTCCATAATGGT
Serotype V Forward	GAGGCCAATCAGTTGCACGTAA
Serotype V Reverse	AACCTTCTCCTTCACACTAATCCT

Table S7. Primers used in this study.

REFERENCES AND NOTES

1. R. Dowey, A. Iqbal, S. R. Heller, I. Sabroe, L. R. Prince, A bittersweet response to infection in diabetes: Targeting neutrophils to modify inflammation and improve host immunity. *Front. Immunol.* **12**, 1–21 (2021).

2. E. Estelle, N. Mathioudakis, Update on management of diabetic foot ulcers. *Ann. N. Y. Acad. Sci.* **176**, 139–148 (2018).

3. J. Davidson, Wound healing. *Adv. Dent. Res.* **9**, 8 (1995).

4. M. A. M. Loots, E. N. Lamme, J. Zeegelaar, J. R. Mekkes, J. D. Bos, E. Middelkoop, Differences in cellular infiltrate and extracellular matrix of chronic diabetic and venous ulcers versus acute wounds. *J. Invest. Dermatol.* **111**, 850–857 (1998).

5. S. A. Eming, T. Krieg, J. M. Davidson, Inflammation in wound repair: Molecular and cellular mechanisms. *J. Invest. Dermatol.* **127**, 514–525 (2007).

6. C. B. Guest, M. J. Park, D. R. Johnson, G. G. Freund, The implication of proinflammatory cytokines in type 2 diabetes. *Front. Biosci.* **13**, 5187–5194 (2008).

7. E. A. Grice, J. A. Segre, Interaction of the microbiome with the innate immune response in chronic wounds. *Adv. Exp. Med. Biol.* **946**, 55–68 (2012).

8. A. J. Singer, R. A. F. Clark, Cutaneous wound healing. *N. Engl. J. Med.* **341**, 738–746 (1999).

9. H. Young, B. Knepper, W. Hernandez, A. Shor, M. Bruntz, C. Berg, C. S. Price, *Pseudomonas aeruginosa*: An uncommon cause of diabetic foot infection. *J. Am. Podiatr. Med. Assoc.* **105**, 125–129 (2015).

10. H. Young, W. Miller, R. Burnham, S. Heard, C. Berg, T. C. Jenkins, How do preoperative antibiotics affect culture yield in diabetic foot infections? *Open Forum Infect. Dis.* **4**, ofx016 (2017).

11. L. R. Kalan, J. S. Meisel, M. A. Loesche, J. Horwinski, I. Soaita, X. Chen, A. Uberoi, S. E. Gardner, E. A. Grice, Strain- and species-level variation in the microbiome of diabetic wounds is associated with clinical outcomes and therapeutic efficacy. *Cell Host Microbe* **25**, 641–655.e5 (2019).

12. R. Serra, R. Grande, L. Butrico, A. Rossi, U. F. Settimio, B. Caroleo, B. Amato, L. Gallelli, S. de Franciscis, Chronic wound infections: The role of *Pseudomonas aeruginosa* and *Staphylococcus aureus*. *Expert Rev. Anti Infect. Ther.* **13**, 605–613 (2015).

13. G. J. Moran, A. Krishnadasan, R. J. Gorwitz, G. E. Fosheim, L. K. McDougal, R. B. Carey, D. A. Talan; EMERGEncy ID Net Study Group, Methicillin-resistant *S. Aureus* infections among patients in the emergency department. *N. Engl. J. Med.* **355**, 666–674 (2006).

14. L. R. Thurlow, A. C. Stephens, K. E. Hurley, A. R. Richardson, Lack of nutritional immunity in diabetic skin infections promotes *Staphylococcus aureus* virulence. *Sci. Adv.* **6**, eabc5569 (2020).

15. R. Jacquet, A. E. LaBauve, L. Akoolo, S. Patel, A. A. Alqarzaee, T. W. F. Lung, K. Poorey, T. P. Stinear, V. C. Thomas, R. J. Meagher, D. Parker, Dual gene expression analysis identifies factors associated with *Staphylococcus aureus* virulence in diabetic mice. *Infect. Immun.* **87**, e00163-19 (2019).

16. K. Gjødsbøl, J. J. Christensen, T. Karlsmark, B. Jørgensen, B. M. Klein, K. A. Krogfelt, Multiple bacterial species reside in chronic wounds : A longitudinal study. *Int. Wound J.* **3**, 225–231 (2006).

17. H. Trøstrup, C. J. Lerche, L. J. Christophersen, K. Thomsen, P. Ø. Jensen, H. P. Hougen, N. Høiby, C. Moser, Chronic *Pseudomonas aeruginosa* biofilm infection impairs murine S100A8/A9 and neutrophil effector cytokines-implications for delayed wound closure? *Pathog. Dis.* **75**, 1–39 (2017).

18. H. Maeda, T. Akaike, Y. Sakata, K. Maruo, Role of Bradykinin in Microbial Infection: Enhancement of Septicemia by Microbial Proteases and Kinin, in *Proteases, Protease Inhibitors and Protease-Derived Peptides* (Birkhäuser Basel, 1993), pp. 159–165.

19. Y. Hidekatsu, Group B *Streptococcus* infection and diabetes: A review. *J. Microbiol. Antimicrob.* **4**, 1–5 (2012).

20. B. Schwartz, A. Schuchat, M. J. Oxtoby, S. L. Cochi, A. Hightower, C. V. Broome, Invasive group B streptococcal disease in adults: A population-based study in Metropolitan Atlanta. *JAMA* **266**, 1112–1114 (1991).

21. J. A. Regan, M. A. Klebanoff, R. P. Nugent, The epidemiology of group B streptococcal colonization in pregnancy. Vaginal infections and prematurity study group. *Obstet. Gynecol.* **77**, 604–610 (1991).

22. H. W. Wilkinson, Group B streptococcal infection in humans. *Annu. Rev. Microbiol.* **32**, 41–57 (1978).

23. J. Gaschignard, C. Levy, O. Romain, R. Cohen, E. Bingen, Y. Aujard, P. Boileau, Neonatal bacterial meningitis. *Pediatr. Infect. Dis. J.* **30**, 212–217 (2011).

24. T. H. Skoff, M. M. Farley, S. Petit, A. S. Craig, W. Schaffner, K. Gershman, L. H. Harrison, R. Lynfield, J. Mohle-Boetani, S. Zansky, B. A. Albanese, K. Stefonek, E. R. Zell, D. Jackson, T. Thompson, S. J. Schrag, Increasing burden of invasive group B streptococcal disease in nonpregnant adults, 1990–2007. *Clin. Infect. Dis.* **49**, 85–92 (2009).

25. M. Rosa-Fraile, S. Dramsi, B. Spellerberg, Group B streptococcal haemolysin and pigment, a tale of twins. *FEMS Microbiol. Rev.* **38**, 932–946 (2014).

26. M. Buscetta, A. Firon, G. Pietrocola, C. Biondo, G. Mancuso, A. Midiri, L. Romeo, R. Galbo, M. Venza, I. Venza, P. A. Kaminski, M. Gominet, G. Teti, P. Speziale, P. Trieu-Cuot, C. Beninati, PbsP, a cell wall-anchored protein that binds plasminogen to promote hematogenous dissemination of group B *Streptococcus*. *Mol. Microbiol.* **101**, 27–41 (2016).

27. K. A. Patras, J. Derieux, M. M. al-Bassam, N. Adiletta, A. Vrbanac, J. D. Lapek, K. Zengler, D. J. Gonzalez, V. Nizet, Group B streptococcus biofilm regulatory protein A contributes to bacterial physiology and innate immune resistance. *J Infect Dis* **218**, 1641–1652 (2018).

28. B. Armistead, E. Oler, K. Adams Waldorf, L. Rajagopal, The double life of group B sStreptococcus: Asymptomatic colonizer and potent pathogen. *J. Mol. Biol.* **431**, 2914–2931 (2019).

29. L. Deng, B. L. Spencer, J. A. Holmes, R. Mu, S. Rego, T. A. Weston, Y. Hu, G. F. Sanches, S. Yoon, N. Park, P. E. Nagao, H. F. Jenkinson, J. A. Thornton, K. S. Seo, A. H. Nobbs, K. S. Doran, The group B streptococcal surface antigen I/II protein, BspC, interacts with host vimentin to promote adherence to brain endothelium and inflammation during the pathogenesis of meningitis. *PLOS Pathog.* **15**, e1007848 (2019).

30. K. M. Edmond, C. Kortsalioudaki, S. Scott, S. J. Schrag, A. K. M. Zaidi, S. Cousens, P. T. Heath, Group B streptococcal disease in infants aged younger than 3 months: Systematic review and meta-analysis. *Lancet* **379**, 547–556 (2012).

31. D. Beier, R. Gross, Regulation of bacterial virulence by two-component systems. *Curr. Opin. Microbiol.* **9**, 143–152 (2006).

32. S.-M. Jiang, M. J. Cieslewicz, D. L. Kasper, M. R. Wessels, Regulation of virulence by a two-component system in group B *Streptococcus*. **187**, 1105–1113 (2005).

33. C. Faralla, M. M. Metruccio, M. de Chiara, R. Mu, K. A. Patras, A. Muzzi, G. Grandi, I. Margarit, K. S. Doran, R. Janulczyk, Analysis of two-component systems in group B *Streptococcus* shows that RgfAC and the novel FspSR modulate virulence and bacterial fitness. *MBio* **5**, 1–12 (2014).

34. M.-C. Lamy, M. Zouine, J. Fert, M. Vergassola, E. Couve, E. Pellegrini, P. Glaser, F. Kunst, T. Msadek, P. Trieu-Cuot, C. Poyart, CovS/CovR of group B *Streptococcus*: A two-component global regulatory system involved in virulence. *Mol. Microbiol.* **54**, 1250–1268 (2004).

35. K. A. Patras, N. Y. Wang, E. M. Fletcher, C. K. Cavaco, A. Jimenez, M. Garg, J. Fierer, T. R. Sheen, L. Rajagopal, K. S. Doran, Group B *Streptococcus* CovR regulation modulates host immune signalling pathways to promote vaginal colonization. *Cell. Microbiol.* **15**, 1154–1167 (2013).

36. A. Lembo, M. A. Gurney, K. Burnside, A. Banerjee, M. de Los Reyes, J. E. Connelly, W. J. Lin, K. A. Jewell, A. Vo, C. W. Renken, K. S. Doran, L. Rajagopal, Regulation of CovR expression in group B *Streptococcus* impacts blood–brain barrier penetration. *Mol. Microbiol.* **77**, 431–443 (2010).

37. M. A. Mandel, A. A. Mahmoud, Impairment of cell-mediated immunity in mutation diabetic mice (db/db). *J. Immunol.* **120**, 1375–1377 (1978).

38. K. P. Hummel, M. M. Dickie, D. L. Coleman, Diabetes, a new mutation in the mouse. *Science* **153**, 1127–1128 (1966).

39. J. M. Edwards, N. Watson, C. Focht, C. Wynn, C. A. Todd, E. B. Walter, R. P. Heine, G. K. Swamy, Group B *Streptococcus* (GBS) colonization and disease among pregnant women: A historical cohort study. *Infect. Dis. Obstet. Gynecol.* **2019**, 1–6 (2019).

40. C. Poyart, A. Tazi, H. Réglier-Poupet, A. Billoët, N. Tavares, J. Raymond, P. Trieu-Cuot, Multiplex PCR assay for rapid and accurate capsular typing of group B streptococci. *J. Clin. Microbiol.* **45**, 1985–1988 (2007).

41. L. Deng, K. Schilcher, L. R. Burcham, J. M. Kwiecinski, P. M. Johnson, S. R. Head, D. E. Heinrichs, A. R. Horswill, K. S. Doran, Identification of key determinants of *Staphylococcus aureus* vaginal colonization. *MBio* **10**, e02321-19 (2019).

42. A. Fabregat, K. Sidiropoulos, G. Viteri, O. Forner, P. Marin-Garcia, V. Arnau, P. D'Eustachio, L. Stein, H. Hermjakob, Reactome pathway analysis: A high-performance in-memory approach. *BMC Bioinformatics* **18**, 1–9 (2017).

43. H. Zhang, B. J. Potter, J. M. Cao, C. Zhang, Interferon-γ–induced adipose tissue inflammation is linked to endothelial dysfunction in type 2 diabetic mice. *Basic Res. Cardiol.* **106**, 1135–1145 (2011).

44. X. Gao, S. Belmadani, A. Picchi, X. Xu, B. J. Potter, N. Tewari-Singh, S. Capobianco, W. M. Chilian, C. Zhang, Tumor necrosis factor-α induces endothelial dysfunction in Lepr db mice. *Circulation* **115**, 245–254 (2007).

45. S. M. Jiang, N. Ishmael, J. D. Hotopp, M. Puliti, L. Tissi, N. Kumar, M. J. Cieslewicz, H́. Tettelin, M. R. Wessels, Variation in the group B *Streptococcus* CsrRS regulon and effects on pathogenicity. *J. Bacteriol.* **190**, 1956–1965 (2008).

46. M. V. Mazzuoli, M. Daunesse, H. Varet, I. Rosinski-Chupin, R. Legendre, O. Sismeiro, M. Gominet, P. A. Kaminski, P. Glaser, C. Chica, P. Trieu-Cuot, A. Firon, The CovR regulatory network drives the evolution of group B *Streptococcus* virulence. *PLOS Genet.* **17**, 1–27 (2021).

47. L. Rajagopal, Understanding the regulation of group B streptococcal virulence factors. *Future Microbiol.* **4**, 201–221 (2009).

48. A. Lupo, C. Ruppen, A. Hemphill, B. Spellerberg, P. Sendi, Phenotypic and molecular characterization of hyperpigmented group B streptococci. *Int. J. Med. Microbiol.* **304**, 717–724 (2014).

49. C. Whidbey, K. Burnside, R. M. Martinez, C. Gendrin, J. Vornhagen, A. Frando, M. I. Harrell, R. M. Adams, L. Rajagopal, A hyperhemolytic/hyperpigmented group B *Streptococcus* strain with a CovR mutation isolated from an adolescent patient with sore throat. *Clin. Res. Infect. Dis.* **2**, 1–12 (2015).

50. K. S. Doran, J. C. W. Chang, V. M. Benoit, L. Eckmann, V. Nizet, Group B streptococcal β-hemolysin/cytolysin promotes invasion of human lung epithelial cells and the release of interleukin-8. *J Infect Dis* **185**, 196–203 (2002).

51. G. Y. Liu, K. S. Doran, T. Lawrence, N. Turkson, M. Puliti, L. Tissi, V. Nizet, Sword and shield: Linked group B streptococcal β-hemolysin/cytolysin and carotenoid pigment function to subvert host phagocyte defense. *Proc. Natl. Acad. Sci. U.S.A.* **101**, 14491–14496 (2004).

52. B. Armistead, P. Herrero-Foncubierta, M. Coleman, P. Quach, C. Whidbey, J. Justicia, R. Tapia, R. Casares, A. Millán, A. Haidour, J. R. Granger, J. Vornhagen, V. Santana-Ufret, S. Merillat, K. A. Waldorf, J. M. Cuerva, L. Rajagopal, Lipid analogs reveal features critical for hemolysis and diminish granadaene mediated group B *Streptococcus* infection. *Nat. Commun.* **11**, 1502 (2020).

53. S. Papasergi, R. Galbo, V. Lanza-Cariccio, M. Domina, G. Signorino, C. Biondo, I. Pernice, C. Poyart, P. Trieu-Cuot, G. Teti, C. Beninati, Analysis of the *Streptococcus agalactiae* exoproteome. *J. Proteomics* **89**, 154–164 (2013).

54. G. Lentini, A. Midiri, A. Firon, R. Galbo, G. Mancuso, C. Biondo, E. Mazzon, A. Passantino, L. Romeo, P. Trieu-Cuot, G. Teti, C. Beninati, The plasminogen binding protein PbsP is required for brain invasion by hypervirulent CC17 group B *Streptococci*. *Sci. Rep.* **8**, 1–11 (2018).

55. G. V. De Gaetano, G. Pietrocola, L. Romeo, R. Galbo, G. Lentini, M. Giardina, C. Biondo, A. Midiri, G. Mancuso, M. Venza, I. Venza, A. Firon, P. Trieu-Cuot, G. Teti, P. Speziale, C. Beninati, The *Streptococcus agalactiae* cell wall-anchored protein PbsP mediates adhesion to and invasion of epithelial cells by exploiting the host vitronectin/αvintegrin axis. *Mol. Microbiol.* **110**, 82–94 (2018).

56. M. A. Dickson, W. C. Hahn, Y. Ino, V. Ronfard, J. Y. Wu, R. A. Weinberg, D. N. Louis, F. P. Li, J. G. Rheinwald, Human keratinocytes that express hTERT and also bypass a p16 INK4a - enforced mechanism that limits life span become immortal yet retain normal growth and differentiation characteristics. *Mol. Cell. Biol.* **20**, 1436–1447 (2000).

57. S. L. Wong, M. Demers, K. Martinod, M. Gallant, Y. Wang, A. B. Goldfine, C. R. Kahn, D. D. Wagner, Diabetes primes neutrophils to undergo NETosis, which impairs wound healing. *Nat. Med.* **21**, 815–819 (2015).

58. F. Hanses, S. Park, J. Rich, J. C. Lee, Reduced neutrophil apoptosis in diabetic mice during staphylococcal infection leads to prolonged TNF-α production and reduced neutrophil clearance. *PLOS ONE* **6**, e23633 (2011).

59. S. G. Jones, R. Edwards, D. W. Thomas, Inflammation and wound healing: The role of bacteria in the immuno-regulation of wound healing. *Int. J. Low. Extrem. Wounds* **3**, 201–208 (2004).

60. J. Wennekamp, P. Henneke, Induction and termination of inflammatory signaling in group B streptococcal sepsis. *Immunol. Rev.* **225**, 114–127 (2008).

61. C. J. Baker, D. L. Kasper, Microcapsule of type III strains of group B *Streptococcus*: Production and morphology. *Infect. Immun.* **13**, 189–194 (1976).

62. T. Sumitomo, M. Nakata, M. Higashino, M. Yamaguchi, S. Kawabata, Group A *Streptococcus* exploits human plasminogen for bacterial translocation across epithelial barrier via tricellular tight junctions. *Sci. Rep.* **6**, 1–8 (2016).

63. Y. Guo, J. Li, E. Hagström, T. Ny, Beneficial and detrimental effects of plasmin(Ogen) during infection and sepsis in mice. *PLOS ONE* **6**, e24774 (2011).

64. Y. Shen, Y. Guo, P. Mikus, R. Sulniute, M. Wilczynska, T. Ny, J. Li, Plasminogen is a key proinflammatory regulator that accelerates the healing of acute and diabetic wounds. *Blood* **119**, 5879–5887 (2012).

65. L. C. C. Cook, H. Hu, M. Maienschein-cline, J. Federle, A vaginal tract signal detected by the Group B *Streptococcus* SaeRS system elicits transcriptomic changes and enhances. *Infect. Immun.* **86**, e00762-17 (2018).

66. K. Kroeze, J. D. D. Pronk, G. Kirtschig, E. D. Boer, E. Middelkoop, R. Scheper, S. Gibbs, Comparison of cytokine, chemokine and growth factor profiles in burn wounds, chronic wounds and surgical excision wounds. (2012).

67. A. Derré-Bobillot, N. G. Cortes-Perez, Y. Yamamoto, P. Kharrat, E. Couvé, V. da Cunha, P. Decker, M. C. Boissier, F. Escartin, B. Cesselin, P. Langella, L. G. Bermúdez-Humarán, P.

Gaudu, Nuclease A (Gbs0661), an extracellular nuclease of *Streptococcus agalactiae*, attacks the neutrophil extracellular traps and is needed for full virulence. *Mol. Microbiol.* **89**, 518–531 (2013).

68. P. Kumar, Wound healing research: Current trends and future directions, V. Kothari, Ed. (Springer, 1 ed. 2021) pp. 704.

69. A. Hotterbeekx, S. Kumar-Singh, H. Goossens, S. Malhotra-Kumar, In vivo and In vitro interactions between *Pseudomonas aeruginosa* and *Staphylococcus* spp. *Front. Cell. Infect. Microbiol.* **7**, 1–13 (2017).

70. D. Pérez-Pascual, P. Gaudu, B. Fleuchot, C. Besset, I. Rosinski-Chupin, A. Guillot, V. Monnet, R. Gardan, RovS and its associated signaling peptide form a cell-to-cell communication system required for *Streptococcus agalactiae* pathogenesis. *MBio* **6**, e02306-14 (2015).

71. L. C. Cook, B. LaSarre, M. J. Federle, Interspecies communication among commensal and pathogenic streptococci. *MBio* **4**, e00382-13 (2013).

72. L. Joubert, J. B. Dagieu, A. Fernandez, A. Derré-Bobillot, E. Borezée-Durant, I. Fleurot, A. Gruss, D. Lechardeur, Visualization of the role of host heme on the virulence of the heme auxotroph *Streptococcus agalactiae*. *Sci. Rep.* **7**, 1–13 (2017).

73. F. A. D. T. G Wagener, H. E. van Beurden, J. W. von den Hoff, G. J. Adema, C. G. Figdor, The heme-heme oxygenase system: A molecular switch in wound healing. *Blood* **102**, 521–528 (2013).

74. P. Sendi, L. Johansson, S. Dahesh, N. M. van Sorge, J. Darenberg, M. Norgren, J. Sjölin, V. Nizet, A. Norrby-Teglund, Bacterial phenotype variants in group B streptococcal toxic shock syndrome. *Emerg. Infect. Dis.* **15**, 223–232 (2009).

75. M. J. Walker, A. Hollands, M. L. Sanderson-Smith, J. N. Cole, J. K. Kirk, A. Henningham, J. D. McArthur, K. Dinkla, R. K. Aziz, R. G. Kansal, A. J. Simpson, J. T. Buchanan, G. S. Chhatwal, M. Kotb, V. Nizet, DNase Sda1 provides selection pressure for a switch to invasive group A streptococcal infection. *Nat. Med.* **13**, 981–985 (2007).

76. P. Sumby, A. R. Whitney, E. A. Graviss, F. R. DeLeo, J. M. Musser, Genome-wide analysis of group A streptococci reveals a mutation that modulates global phenotype and disease specificity. *PLOS Pathog.* **2**, e5 (2006).

77. J. J. Velarde, M. Ashbaugh, M. R. Wessels, The human antimicrobial peptide LL-37 binds directly to CsrS, a sensor histidine kinase of group a *Streptococcus*, to activate expression of virulence factors. *J. Biol. Chem.* **289**, 36315–36324 (2014).

78. M. I. Love, W. Huber, S. Anders, Moderated estimation of fold change and dispersion for RNA-seq data with DESeq2. *Genome Biol.* **15**, 1–21 (2014).

79. A. Liberzon, A. Subramanian, R. Pinchback, H. Thorvaldsdóttir, P. Tamayo, J. P. Mesirov, Molecular signatures database (MSigDB) 3.0. *Bioinformatics* **27**, 1739–1740 (2011).

80. B. L. Spencer, L. Deng, K. A. Patras, Z. M. Burcham, G. F. Sanches, P. E. Nagao, K. S. Doran, Cas9 contributes to group B streptococcal colonization and disease. *Front. Microbiol.* **10**, 1–15 (2019).

81. A. R. Flores, J. Galloway-Peña, P. Sahasrabhojane, M. Saldaña, H. Yao, X. Su, N. J. Ajami, M. E. Holder, J. F. Petrosino, E. Thompson, I. Margarit Y Ros, R. Rosini, G. Grandi, N. Horstmann, S. Teatero, A. McGeer, N. Fittipaldi, R. Rappuoli, C. J. Baker, S. A. Shelburne, Sequence type 1 group B *Streptococcus*, an emerging cause of invasive disease in adults, evolves by small genetic changes. *Proc. Natl. Acad. Sci. U.S.A.* **112**, 6431–6436 (2015).

82. L. C. Madoff, J. L. Michel, D. L. Kasper,A monoclonal antibody identifies a protective C-protein alpha-antigen epitope in group B streptococci. *Infect. Immun.* **59**, 204–210 (1991).

83. C. B. Wilson, W. M. Weaver, Comparative susceptibility of group B *Streptococci* and *Staphylococcus aureus* to killing by oxygen metabolites. *J Infect Dis* **152**, 323–329 (1985).
